# PAFAH1B3 Expression Is Correlated With Gastric Cancer Cell Proliferation and Immune Infiltration

**DOI:** 10.3389/fonc.2021.591545

**Published:** 2021-02-25

**Authors:** Tianyu Xie, Xin Guo, Di Wu, Shuo Li, Yixun Lu, Xinxin Wang, Lin Chen

**Affiliations:** ^1^ School of Medicine, Nankai University, Tianjin, China; ^2^ Department of General Surgery, Chinese PLA General Hospital, Beijing, China

**Keywords:** PAFAH1B3, gastric cancer, cell proliferation, immune infiltration, oncogene

## Abstract

**Background:**

Platelet activating factor acetylhydrolase 1b catalytic subunit 3 (PAFAH1B3) is associated with a variety of human diseases. However, its function in gastric cancer remains uncertain.

**Methods:**

PAFAH1B3 expression was analyzed in The Cancer Genome Atlas (TCGA) and genotype-tissue expression pan-cancer data. The association between PAFAH1B3 expression and patient prognosis was evaluated using TCGA clinical survival data. Enrichment analysis of PAFAH1B3 was performed using the *clusterProfiler* R software package. Moreover, the correlation between PAFAH1B3 expression and immune cell infiltration were evaluated by analyzing TCGA database. CCK8 assay and colony-formation assay were performed to assess the effect of PAFAH1B3 on the proliferation of gastric cancer cells. Transwell assay was used to evaluate the impact of PAFAH1B3 on gastric cancer cell migration. Western blot was performed to evaluate the role of PAFAH1B3 on signaling pathways in gastric cancer cells.

**Results:**

PAFAH1B3 was highly expressed in many types of tumors including gastric cancer. High PAFAH1B3 expression was significantly correlated with proliferation-related gene sets involved in DNA replication, the cell cycle, and cell cycle checkpoints. Further analysis showed that high PAFAH1B3 expression was associated with high M1 macrophage and CD8-positive T cell infiltration scores. PAFAH1B3 knockdown inhibited the proliferation, migration, and the activation of oncogenic signaling in gastric cancer cells.

**Conclusions:**

Our findings suggest that PAFAH1B3 may be an oncogene in gastric cancer.

## Introduction

Gastric cancer is a major malignant tumor of the digestive system. It is prone to relapse and metastasis during the late stage and has a high mortality rate (1). At present, there is a lack of effective early diagnostic markers to detect and treat gastric cancer. With the rapid development of high-throughput sequencing technology and transcriptomic research, more driver genes continue to be discovered. However, there remains an urgent need to identify more key driver oncogenes, especially those that affect the composition of the immune microenvironment in gastric cancer.

Platelet activating factor acetylhydrolase 1b catalytic subunit 3 (PAFAH1B3) is one of the catalytic subunits of Platelet-activating factor (PAF) acetylhydrolase, which was reported to play crucial roles in certain types of cancers by regulating PAF activity (2, 3). Previous studies have indicated the association between PAFAH1B3 and cancer progression. For example, PAFAH1B3 expression was found to be higher in hypopharyngeal squamous cell carcinoma tissues than in adjacent non-tumor samples and predicted a poor outcome (4). Selective inhibition of PAFAH1B3 has been reported to impair cancer cell survival (5). However, the role of PAFAH1B3 in gastric cancer remains unknown.

In this study, we evaluated PAFAH1B3 expression in various tumors in The Cancer Genome Atlas (TCGA) and its correlation with patient prognosis. We further examined the association of PAFAH1B3 expression with molecular pathways in gastric cancer. Since immune cell infiltration is important for the prognosis of gastric cancer patients (6, 7), we also examined the correlation between PAFAH1B3 expression and the immune cell infiltration scores. Finally, we examined the effect of PAFAH1B3 knockdown on the apoptosis and proliferation of gastric cancer cells. Our results offer novel insights into the functional role of PAFAH1B3 in gastric cancer.

## Materials and Methods

### Data Collection and Analysis

PAFAH1B3 expression profiles and TCGA and Genotype-Tissue Expression (GTEx) clinical pan-cancer data were downloaded from the University of California, Santa Cruz (UCSC) Xena database (https://xenabrowser.net/datapages/), including data on adrenocortical carcinoma (ACC), bladder urothelial carcinoma (BLCA), breast invasive carcinoma (BRCA), cervical squamous cell carcinoma and endocervical adenocarcinoma (CESC), cholangiocarcinoma (CHOL), colon adenocarcinoma (COAD), esophageal carcinoma (ESCA), glioblastoma multiforme (GBM), head and neck squamous cell carcinoma (HNSC), kidney chromophobe, kidney renal clear cell carcinoma, kidney renal papillary cell carcinoma (KIRP), acute myeloid leukemia (LAML), brain lower grade glioma (LGG), liver hepatocellular carcinoma (LIHC), lung adenocarcinoma (LUAD), lung squamous cell carcinoma (LUSC), ovarian serous cystadenocarcinoma (OV), pancreatic adenocarcinoma (PAAD), prostate adenocarcinoma (PRAD), rectum adenocarcinoma (READ), skin cutaneous melanoma (SCKM), stomach adenocarcinoma (STAD), testicular germ cell tumor (TGCT), thyroid carcinoma (THCA), uterine corpus endometrial carcinoma (UCEC), and uterine carcinosarcoma (UCS). To evaluate PAFAH1B3 expression, tumor tissues were obtained from TCGA, and normal tissues were obtained from TCGA and the GTEx database.

### Correlation and Enrichment Analyses

Pearson correlation analysis of PAFAH1B3 mRNA and other mRNAs was performed in gastric cancer using TCGA STAD data. The 300 genes most positively associated with PAFAH1B3 were selected for enrichment analysis to determine the function of PAFAH1B3. Gene ontology (GO) analysis was performed using the EnrichGO function in the *clusterProfiler* R software package R with the following parameters: *ont* = all, *pvalue-Cutoff* = 0.05, and *qvalue-Cutoff* = 0.05. Gene set enrichment analysis was performed using the gseKEGG and gsePathway functions in *clusterProfiler* with the following parameters: *nPerm* = 1,000, *minGSSize* = 10, *maxGSSize* = 1,000, and *pvalue-Cutoff* = 0.05.

### Immune Cell Infiltration

We downloaded pan-cancer immune cell infiltration scores from TCGA that were derived from a previously published study (8), the results of which were based on the CIBERSORT analytical tool (9). TCGA gastric cancer samples were divided into two groups according to the median PAFAH1B3 expression (high versus low level), and their immune cell infiltration levels were compared.

### Cell Culture and Treatment

AGS, MGC-803, BGC-823, SGC-7901 cell lines were purchased from the Cell Bank of the Shanghai Institute of Cells, Chinese Academy of Science (Shanghai, China). All cell lines were cultured in Dulbecco’s modified Eagle’s medium (Gibco) in 10-cm petri dish (Corning) at 37°C with 5% CO_2_. Cell transfection was performed using the Lipofectamine 2000 reagent (Invitrogen) according to the manufacturer’s protocol. Briefly, cells were seeded in six-well plates (Corning) and grown to a cell density of 30% and then transfected and cultured at 37°C for a further 48 h, followed by harvesting for quantitative reverse-transcription polymerase chain reaction (qRT-PCR) and other experiments.

### RNA Extraction and qRT-PCR

Total RNA from approximately 1×10^6^ cells were isolated using TRIzol reagent (Pufei, Shanghai, China) according to the manufacturer’s protocol. The primers used for qRT-PCR, including those for PAFAH1B3 and GAPDH, were obtained from Applied Biosystems (Ribo, Guangzhou, China). The primer sequences were (5’-3’): PAFAH1B3 forward -GAGAAGAACCGACAGGTGAAC, reverse-CGGCAAACAGGTGTGTAGC. GAPDH forward-CTGGGCTACACTGAGCACC, reverse-AAGTGGTCGTTGAGGGCAATG. QRT-PCR parameters were: 95°C 6 min; (95°C 10 s, 58°C 30 s) × 40 amplification cycles. Relative expression levels were normalized to internal controls and calculated according to the 2–ΔΔCT method.

### Western Blotting

Total protein from approximately 1×10^6^ cells were extracted using radioimmunoprecipitation assay buffer and then quantified using the bicinchoninic acid method (Beyotime, Shanghai, China). Equal amounts of protein sample were separated using 8–15% sodium dodecyl sulfate–polyacrylamide gel electrophoresis and then transferred to nitrocellulose membranes. Membranes were blocked with 5% non-fat milk in TBST (Abcam, Cambridge, UK) for 1 h at room temperature and incubated with primary antibody (Abcam) at 4°C overnight. Membranes were then washed three times andincubated with horseradish peroxidase-labeled secondary antibody (Santa Cruz Biotechnology, Dallas, TX, USA) for 1 h. Signals were detected using an enhanced Chemiluminescent Western Blot Analysis Kit (Thermo Fisher Scientific, Inc.).

### CCK8 Assay

Cells in the logarithmic growth phase from each experimental group were trypsinized, resuspended in complete medium, and cultured overnight. On the second day, cell proliferation was evaluated by Cell Counting Kit-8 reagent (Abcam) according to the manufacturer’s protocol. Optical density values at 460 nm were detected using a microplate reader (Molecular Devices, Rockford, IL, USA).

### Colony-Formation Assay

One-hundred gastric cancer cells were plated into six-well plates and cultured for 10 days. Cell colonies were fixed and stained with crystal violet (Beyotime, China) for 5 min in 10% ethanol. Cell colonies were imaged and counted.

### Transwell Assay

Thirty thousand gastric cancer cells were plated in the upper chamber of the transwell chamber (Corning, USA) in a 24-well plate. 24 h after incubation at 37°C, cells on the top surface of the chamber were removed by wiping. Cells on the bottom surface of the chamber were fixed with 4% paraformaldehyde for 10 min and stained with crystal violet (Beyotime, China) for 5 min. The number of migrated cells were imaged and counted.

### Statistical Analyses

Data are expressed as means ± standard deviation. All data were analyzed using SPSS ver. 21.0 software (SPSS, Inc., Chicago, IL, USA). The normality of the data was tested using the Kolmogorov–Smirnov test. Pairwise differences between groups were analyzed using Student’s *t*-test. Significance was assessed at a level of P < 0.05.

## Results

### Pan-Cancer PAFAH1B3 Expression Analysis

**Figure 1:**
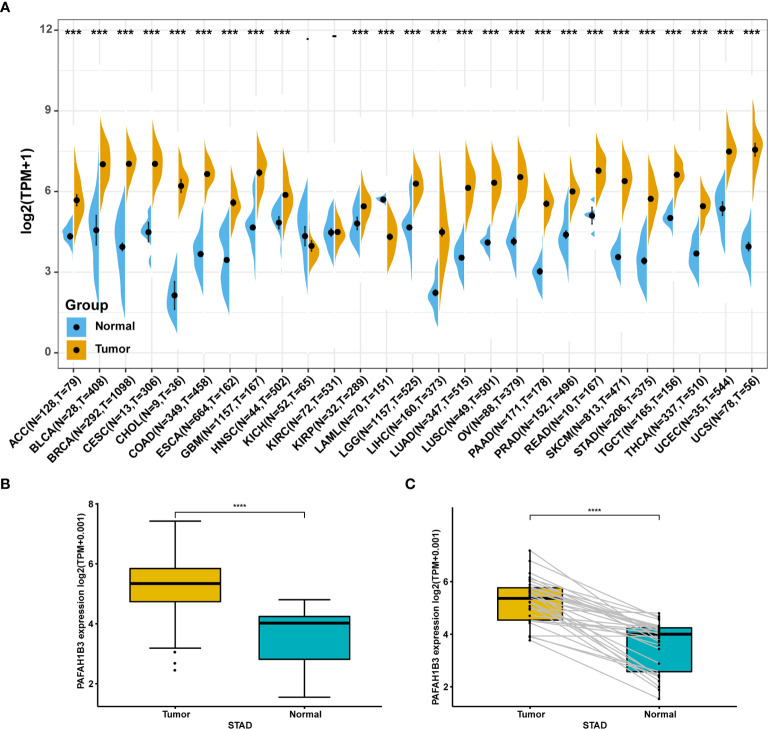
Pan-cancer PAFAH1B3 expression analysis. **(A)** PAFAH1B3 expression in tumor and normal tissues in pan-cancer data of The Cancer Genome Atlas (TCGA) and GTEx. **(B)** PAFAH1B3 expression in tumor and normal tissues in STAD from TCGA. **(C)** PAFAH1B3 expression in paired tumor and normal tissues in STAD from TCGA. Data were shown as mean ± SD. *p < 0.05, **p < 0.01, ***p < 0.001, ****p < 0.0001.

We first evaluated PAFAH1B3 expression in TCGA and GTEx pan-cancer database, and found higher PAFAH1B3 expression in 24 tumors compared with the corresponding normal tissues, including: ACC, BLCA, BRCA, CESC, CHOL, COAD, ESCA, GBM, HNSC, KIRP, LGG, LIHC, LUAD, LUSC, OV, PAAD, PRAD, READ, SCKM, STAD, TGCT, THCA, UCEC, and UCS (**Figure 1A**).

Particularly, high PAFAH1B3 expression was observed in STAD gastric cancer in the TCGA cohort (**Figure 1B**) compared with the adjacent tissues (**Figure 1C**), suggesting that PAFAH1B3 may play a role in the pathogenesis of gastric cancer.

### Association Between PAFAH1B3 Expression and Cancer Patient Prognosis

**Figure 2:**
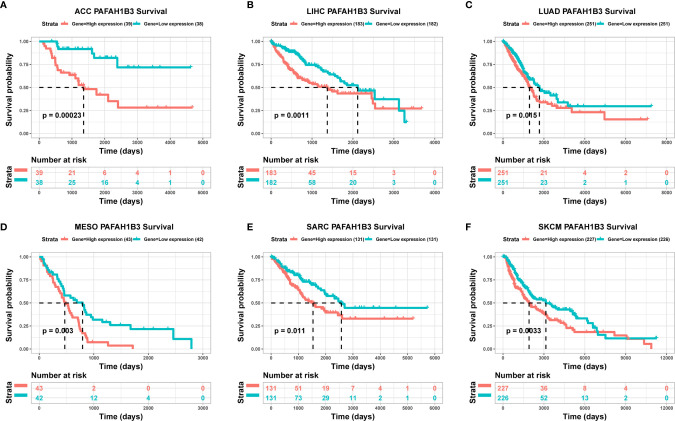
The association between PAFAH1B3 expression and cancer patient prognosis. **(A–F)** The correlation between PAFAH1B3 expression and the prognosis of various cancer types was analyzed using The Cancer Genome Atlas (TCGA) database.

To evaluate the utility of PAFAH1B3 expression in predicting cancer patient prognosis, we analyzed the association between PAFAH1B3 expression and overall survival in the TCGA cohort. The results showed that higher PAFAH1B3 expression was significantly associated with poor prognosis in ACC (P = 0.00023), LIHC (P = 0.0011), LUAD (P = 0.015), MESO (P = 0.003), SARC (P = 0.011), and SKCM (P = 0.0033) (**Figures 2A–F**), indicating that PAFAH1B3 is a potential oncogene in these types of cancers.

### Correlation and Enrichment Analyses

**Figure 3:**
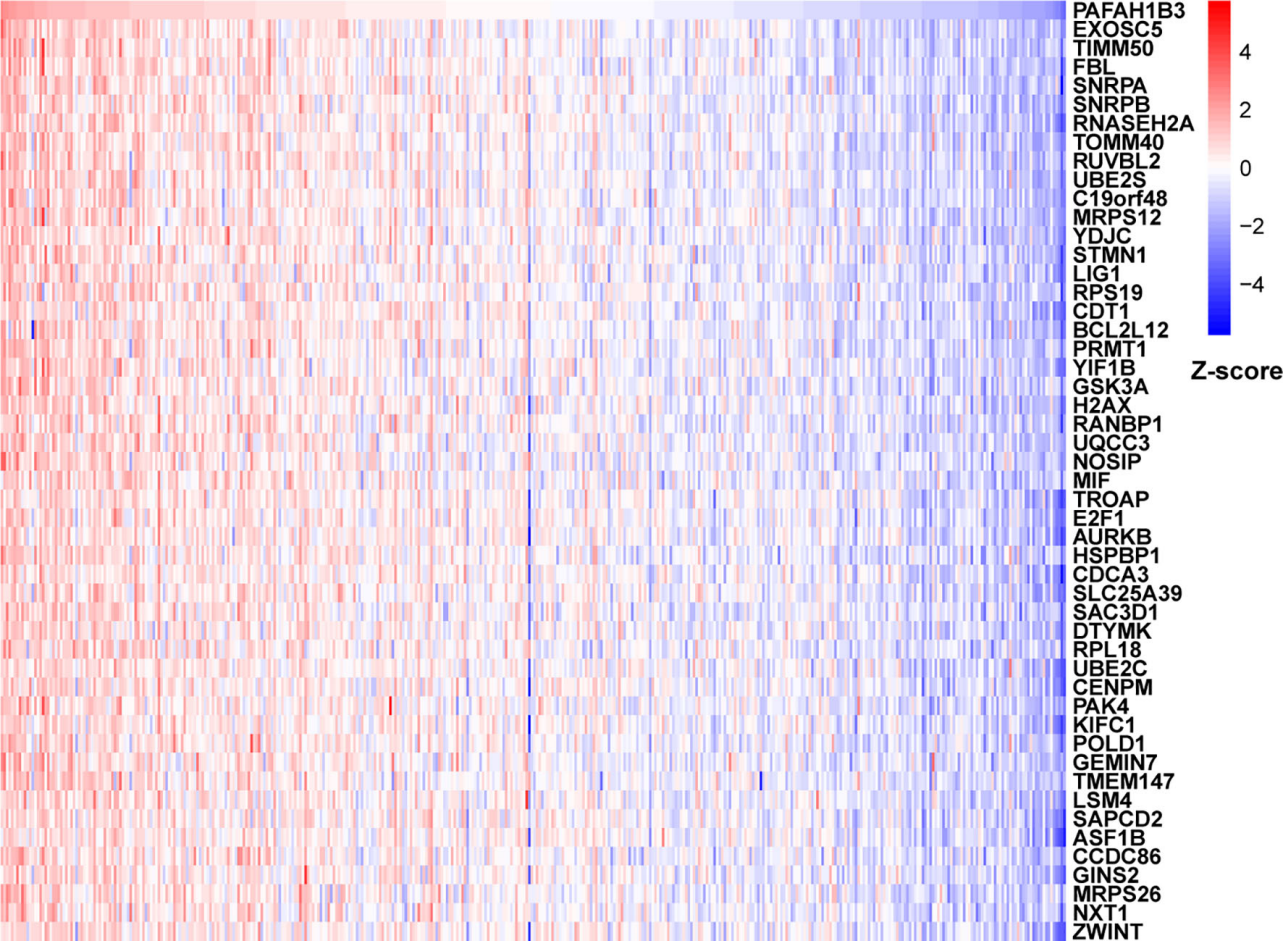
The correlation analysis of PAFAH1B3. Top 50 genes most positively associated with PAFAH1B3 were shown in heatmap. Data were normalized by Z-score standardization method.

**Figure 4:**
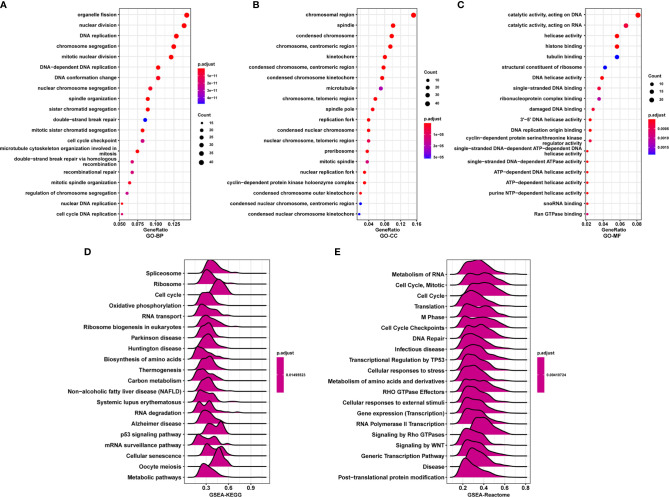
Enrichment analysis of PAFAH1B3 in gastric cancer. **(A–C)** Significant Gene Ontology terms of top 300 genes most positively associated with PAFAH1B3, including biological processes **(A)**, cell component **(B)**, and molecular function **(C)**. **(D, E)** Significant gene set enrichment analysis (GSEA) results of PAFAH1B3, including KEGG pathways **(D)** and Reactome pathways **(E)**.

To further explore the functions and pathways affected by PAFAH1B3, we performed correlation analyses between PAFAH1B3 and all other mRNAs in gastric cancer using TCGA data. The 300 genes most positively associated with PAFAH1B3 were selected for enrichment analysis, and the top 50 genes were displayed in a heatmap (**Figure 3**). We further explored potential functional pathways based on the top 300 genes using the R software *clusterProfiler* package. GO functional enrichment analysis revealed that PAFAH1B3 was associated mainly with cell proliferation-related pathways including DNA replication and cell cycle checkpoints (**Figures 4A–C**). Gene set enrichment analysis was conducted to search the Kyoto Encyclopedia of Genes and Genomes (KEGG) and Reactome pathway databases. The KEGG analysis results showed that spliceosome, ribosome, and cell cycle terms were significantly enriched (**Figure 4D**). The Reactome analysis revealed significant enrichment of cell cycle, M phase, and DNA repair pathways (**Figure 4E**). These results suggest that high PAFAH1B3 expression is associated with the hyperactivation of multiple oncogenic pathways in gastric cancer, especially signalings which control cell proliferation.

### Correlation Between Immune Cell Infiltration and PAFAH1B3 Expression

**Figure 5:**
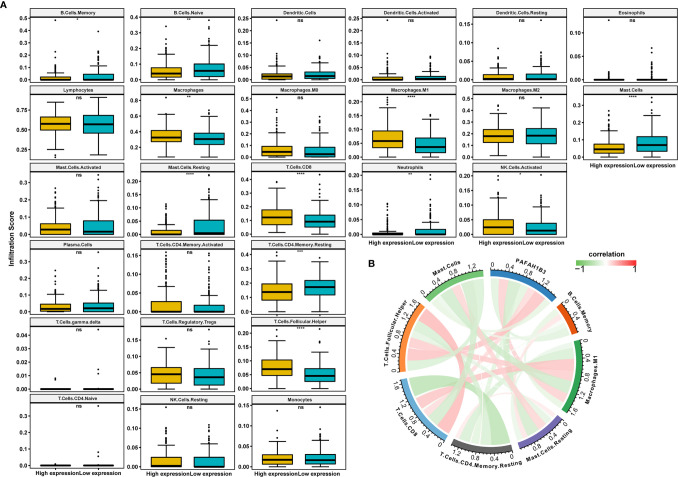
The correlation analysis between immune cell infiltration and PAFAH1B3 in gastric cancer. **(A)** The immune cell infiltration level in high and low PAFAH1B3 expression groups in gastric cancer of The Cancer Genome Atlas (TCGA) cohort. **(B)** The correlation between PAFAH1B3 and the immune cell infiltration levels, red represents positive correlation, green represents negative correlation, and the deeper the color, the stronger the correlation. Data were shown as mean ± SD. *p < 0.05, **p < 0.01, ***p < 0.001, ****p < 0.0001. ns, no significance.

Next, we analyzed the immune cell infiltration scores of gastric cancer patients from TCGA database. The infiltration scores of M1 macrophages and the CD8-positive T cells were higher in the high-PAFAH1B3-expression cohort compared with those in the low-PAFAH1B3-expression cohort (**Figure 5A**).

PAFAH1B3 expression was significantly positively correlated with the M1 macrophage infiltration and CD8-positive T cell infiltration (**Figure 5B**), indicating that high PAFAH1B3 expression promotes the intratumoral accumulation of CD8-positive T cells and macrophages, especially M1-like macrophages. These results suggest that high PAFAH1B3 expression is closely related to the immune-activated status of gastric cancer.

### PAFAH1B3 Knockdown Inhibited the Malignant Behaviors and Signaling in Gastric Cancer Cells

**Figure 6:**
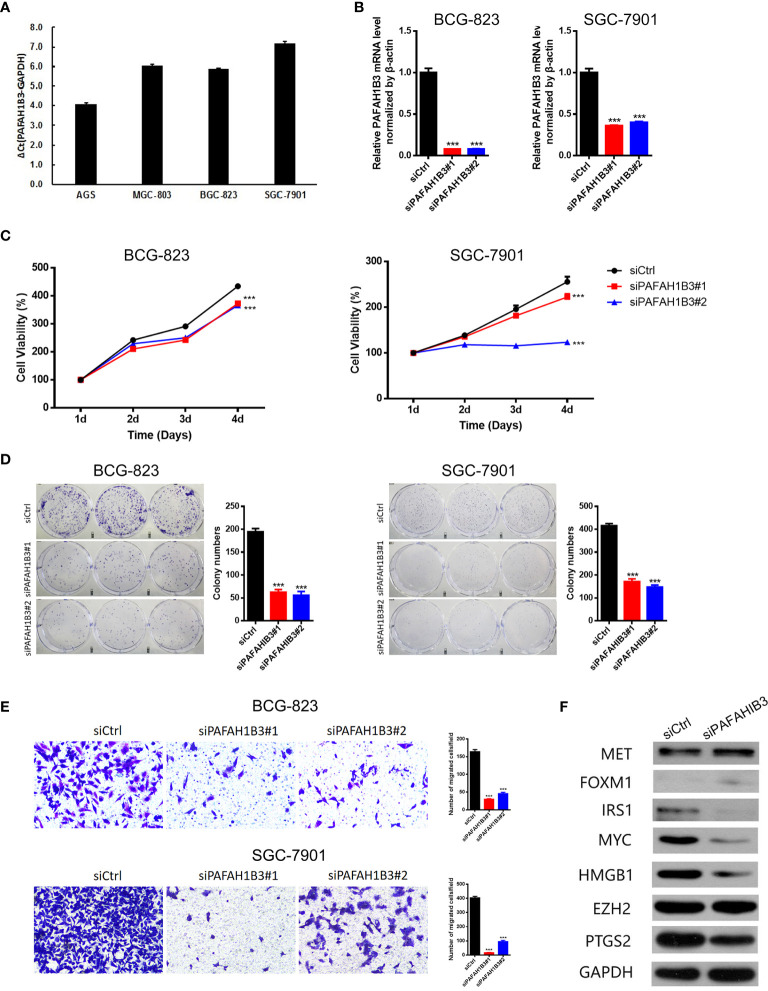
Knockdown of PAFAH1B3 inhibited the proliferation and migration of gastric cancer cells. **(A)** The level of PAFAH1B3 was evaluated in various gastric cancer cell lines by qRT-PCR. **(B)** BCG-823 and SGC-7901 cells were transfected with si-PAFAH1B3, the level of PAFAH1B3 was evaluated by qRT-PCR. **(C, D)** The proliferation of gastric cells (GC) was examined by CCK-8 assay **(C)** and colony-formation assay. **(E)** The migration of GC cells was examined by transwell assay. **(F)** The expression levels of indicated proteins were evaluated by Western blot in control and PAFAH1B3-silenced BCG-823 cells. Data were shown as mean ± SD. ***p < 0.001.

Next, we evaluated PAFAH1B3 expression in multiple gastric cancer cell lines, and found relatively higher expression of PAFAH1B3 in BGS-823 and SGC-7901 cells compared with that in AGS and MGS-803 cells (**Figure 6A**). To explore the biological effects of PAFAH1B3 on gastric cancer cell proliferation, we knocked down PAFAH1B3 expression in BGS-823 and SGC-7901 cells *via* two PAFAH1B3 shRNA, and validated the successful silence of PAFAH1B3 expression in these two cell lines (**Figure 6B**). Next, we conducted CCK8 assay to evaluate cell proliferation. The Results showed that the proliferation rates of the BGS-823 and SGC-7901 cells were significantly inhibited following PAFAH1B3 knockdown (**Figure 6C**). The negative effect of PAFAH1B3 on gastric cancer cell proliferation was further confirmed by colony-formation assay (**Figure 6D**). Besides, PAFAH1B3 silencing also significantly impaired the migratory ability of gastric cancer cells as evidenced by Transwell assay (**Figure 6E**). Finally, we looked at the impact of PAFAH1B3 on several oncogenic signaling pathways by Western blot. The results showed that PAFAH1B3 knockdown in SGC-7901 cells led to the obviously downregulated levels of IRS1, Myc, HMGB1, and PTGS2. On the other hand, the levels of MET and FOXM1 were slightly upregulated. The level of EZH2 was unchanged by PAFAH1B3 knockdown (**Figure 6F**). Taken together, PAFAH1B3 facilitates the proliferation, migration, and the activation of oncogenic signaling in gastric cancer cells.

## Discussion

In recent years, PAFAH1B3 was reported to play regulatory roles in various kinds of diseases, including human cancers. For example, in hypopharyngeal squamous cell carcinoma (HSCC), the expression of PAFAH1B3 was reported to be upregulated and was correlated with poor patient prognosis. Silencing PAFAH1B3 expression restricted the proliferation, invasion, and migration of HSCC cells (4). Metabolomic study indicated that inhibition of PAFAH1B3 suppressed the growth of breast cancer by upregulating the levels of anti-tumor lipids, such as ceramides and several PPARα ligands, indicating that PAFAH1B3 serves as a metabolic oncogene in breast cancer (10). Similarly, selective small-molecule PAFAH1b3 inhibitors impaired the growth of neuroblastoma cells, suggesting that PAFAH1b3 might become a targetable oncoprotein (5). Nevertheless, the expression pattern of PAFAH1B3 in various human cancers and its prognostic values are still elusive. Here, by data mining using TCGA datasets, the hyperexpression of PAFAH1B3 was observed in 24 types of tumor tissues compared with normal tissues, suggesting that PAFAH1B3 might serve as a novel oncogene in these tumor types.

Next, we examined the expression level of PAFAH1B3 and its prognostic value using pan-cancer TCGA and GTEx data obtained from the UCSC Xena database. Compared with normal tissues, we found that PAFAH1B3 was highly expressed in ACC, BLCA, BRCA, CESC, CHOL, COAD, ESCA, GBM, HNSC, KIRP, LGG, LIHC, LUAD, LUSC, OV, PAAD, PRAD, READ, SCKM, STAD, TGCT, THCA, UCEC, and UCS, whereas its expression was low in LAML tissue. PAFAH1B3 was particularly highly expressed in gastric cancer tissues compared with adjacent normal tissues according to TCGA data. Overexpression of PAFAH1B3 generally predicts a poor prognosis in ACC, LIHC, LUAD, MESO, SARC, and SKCM. These results support the use of PAFAH1B3 as a prognostic biomarker of tumor prognosis.

Our enrichment analysis results showed that PAFAH1B3 is closely related to cell proliferation-related pathways such as DNA replication, the cell cycle, and cell cycle checkpoints. Further analyses demonstrated that PAFAH1B3 knockdown inhibited the proliferation of the AGS and MGC-803 gastric cancer cell lines. On the other hand, the signaling mechanisms underlying the function of PAFAH1B3 in tumor cells are largely unknown to date. Here, we found that PAFAH1B3 knockdown in gastric cancer cells downregulated the levels of IRS1, Myc, HMGB1, and PTGS2, which were reported to serve as oncogenes in many types of cancers (11–14). These results suggest that PAFAH1B3 facilitated the activation of multiple oncogenic signaling pathways in gastric cancer cells.

Stroma cells in tumor microenvironment, especially immune cells, are vital elements which have profound impacts on regulating the malignant behaviors of tumor cells (15–17). Accumulating evidence has elucidated their clinicopathological significance in predicting the outcomes and therapeutic efficacy in cancer patients (18, 19). The infiltration of CD8-positive T cells and tumor-associated macrophages was reported to facilitate the progression of epithelial gastric cancer (20–22). In the present study, infiltration levels of CD8-positive T cells and M1 macrophages were significantly higher in the gastric cancer group with high PAFAH1B3 expression. Moreover, positive correlations were detected between PAFAH1B3 expression and infiltration levels of CD8-positive T cells and M1 macrophages, indicating a key role of PAFAH1B3 in regulating tumor immunology.

In summary, PAFAH1B3 may play a pathogenic role in gastric cancer progression, both acting on tumor cells or on tumor-infiltrating immune cells.

## Data Availability Statement

The datasets presented in this study can be found in online repositories. The names of the repository/repositories and accession number(s) can be found in the article/supplementary material.

## Author Contributions

LC and XW designed the research. TX, DW, and SL performed the research and collected the data. XG and YL analyzed the data. TX drafted the manuscript. All authors contributed to the article and approved the submitted version.

## Funding

National Defense Science and Technology Strategy Pilot program No. 19-ZLXD-12-35-03-600-01; Clinical Research Support Fund of PLA General Hospital No 2019XXJSYX07.

## Conflict of Interest

The authors declare that the research was conducted in the absence of any commercial or financial relationships that could be construed as a potential conflict of interest.
